# Large-scale high uniform optoelectronic synapses array for artificial visual neural network

**DOI:** 10.1038/s41378-024-00859-2

**Published:** 2025-01-13

**Authors:** Fanqing Zhang, Chunyang Li, Zhicheng Chen, Haiqiu Tan, Zhongyi Li, Chengzhai Lv, Shuai Xiao, Lining Wu, Jing Zhao

**Affiliations:** 1 State Key Laboratory of Explosion Science and Safety Protection, Beijing Institute of Technology, Ministry of Education, 100081 Beijing, China; 2 School of Mechatronical Engineering, Beijing Institute of Technology, 100081 Beijing, China; 3 Beijing Advanced Innovation Center for Intelligent Robots and Systems, Beijing Institute of Technology, 100081 Beijing, China; 4 Laser Micro/Nano Fabrication Laboratory, School of Mechanical Engineering, Beijing Institute of Technology, 100081 Beijing, China; 5 School of Mechanical Engineering, Beijing Institute of Technology, 100081 Beijing, China

**Keywords:** Electronic properties and materials, Electronic devices, Electronic properties and materials

## Abstract

Recently, the biologically inspired intelligent artificial visual neural system has aroused enormous interest. However, there are still significant obstacles in pursuing large-scale parallel and efficient visual memory and recognition. In this study, we demonstrate a 28 × 28 synaptic devices array for the artificial visual neuromorphic system, within the size of 0.7 × 0.7 cm^2^, which integrates sensing, memory, and processing functions. The highly uniform floating-gate synaptic transistors array were constructed by the wafer-scale grown monolayer molybdenum disulfide with Au nanoparticles (NPs) acting as the electrons capture layers. Various synaptic plasticity behaviors have been achieved owing to the switchable electronic storage performance. The excellent optical/electrical coordination capabilities were implemented by paralleled processing both the optical and electrical signals the synaptic array of 784 devices, enabling to realize the badges and letters writing and erasing process. Finally, the established artificial visual convolutional neural network (CNN) through optical/electrical signal modulation can reach the high digit recognition accuracy of 96.5%. Therefore, our results provide a feasible route for future large-scale integrated artificial visual neuromorphic system.

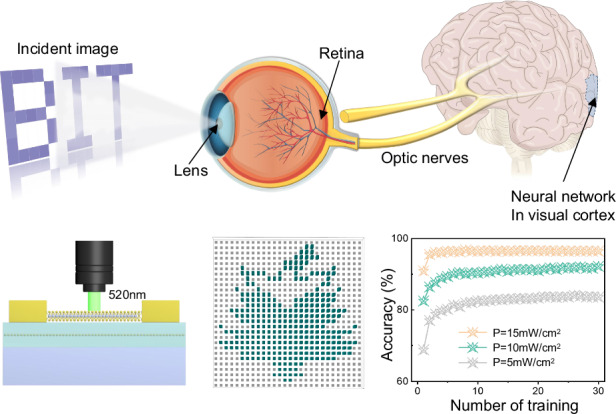

## Introduction

In the human visual system, information acquisition and processing are carried out within the same framework. However, current advancements in neuromorphic vision technology are impeded by challenges such as high circuit complexity, increased power consumption, low efficiency, and device miniaturization, primarily due to the physical separation between signal devices and processing units. Prior studies indicate that employing a single device to simulate the biomimetic vision systems falls short of the requirements for parallel processing of visual information^1–10^. Therefore, there is a pressing need for biologically inspired strategies, such as the smarter visual perception and computational processing design solutions^11^. The biological visual system has its natural advantages, optical information perceived by the eyes is transmitted between neurons through synapses, ultimately reaching the neural network in the cerebral cortex for further processing and memory learning. Synapses characterized by low-power consumption (approximately 10 fJ per synaptic event), high speed and parallelism can surpass the properties of the supercomputers^12–26^. As a result, the rapid development of the bio-inspired optoelectronic synapses that combine light detection and synaptic plasticity learning capabilities is necessary to achieve an advanced artificial vision system with intrinsic visual perception and neuromorphic computational behavior.

Recently, transition metal dichalcogenides (TMDs) have shone brightly in semiconductor materials. Especially, single-layer MoS_2_ is an emerging two-dimensional (2D) TMDs material with great potential in future artificial intelligence electronic devices due to its excellent electrical and optical properties, which has aroused great interest^26–33^. Moreover, its extraordinarily high surface-to-volume ratio enhances the device’s sensitivity to charge presence^34–36^. Previously, artificial synaptic devices based on 2D materials and their heterostructures only focus on a single device as a-proof-of-concept, and large-scale synaptic devices array was rarely investigated, which was restricted by the size of the materials^5,37–41^. Furthermore, the design and manufacturing of 2D synaptic devices usually involve complex processes, especially the floating-gate devices, posing challenges to achieving highly uniform and scalable synaptic arrays. Unlike heterojunction transistors, which start from the perspective of channel materials, floating-gate transistors aim to achieve storage performance through substrates. And the high-k material HfO_2_ was used to adjust devices with lower gate voltage, which is expected to achieve lower power consumption^33,42–48^. Nonetheless, it is necessary to promote the development of artificial neural networks (ANNs) through constructing large-scale high-density integrated synaptic devices array to simulate the function of the biological vision at the hardware scale. Large-scale 2D material integrated device arrays based on floating-gate transistors can simulate biological vision systems from mechanism to effect, promoting the development of artificial vision neural networks.

In this article, we report a highly uniform artificial visual neural network based on a wafer-scale monolayer MoS_2_ floating-gate field-effect transistors array (28 × 28 devices, 0.7 × 0.7 cm^2^), enabling the integrated application for simulating human visual neural networks. According to the Au NPs floating-gate layer capture and slowly release charges, thereby effectively realizing synaptic plasticity, including EPSC and PPF through the single device. Immediately after, the artificial synaptic device simulated the biomimetic processes of visual signal perception, memory, and processing functions. After testing and analyzing 784 devices, the uniform performance of the devices array revealed a stable on/off rate of ~10^6^ and the mobility of ~8 cm^2^V^−1^s^−1^, highlighting the exceptional optoelectronic synaptic performance. In addition, by applying optical spikes to the device array, the emblem of Beijing Institute of Technology was successfully encoded into a 28 × 28 synaptic devices array. The ability to preserve image information of badges over a long period of time is attributed to the slow discharge of floating gate layer charges. By using an array of optical pulse scanning devices, we were able to sequentially program letter images into the device array, demonstrating the array’s ability to fast write and erase process, and the ability to process photo/electric signals in parallel of the device. Finally, the fabricated artificial synaptic neural network, based on the devices array, leverages both light and electrical spikes for synaptic weight adjustments, achieving a high digit recognition accuracy of 96.5%, proving the network’s potential for image recognition applications. The exceptional performance of the integrated artificial synapses array based on MoS_2_ devices marks a significant stride towards practical applications within a device-to-system level simulation framework, and heralds promising prospects for applications in brain-inspired learning and memory, artificial visual nervous systems, and neural morphology computing.

## Results and discussion

**Fig. 1:**
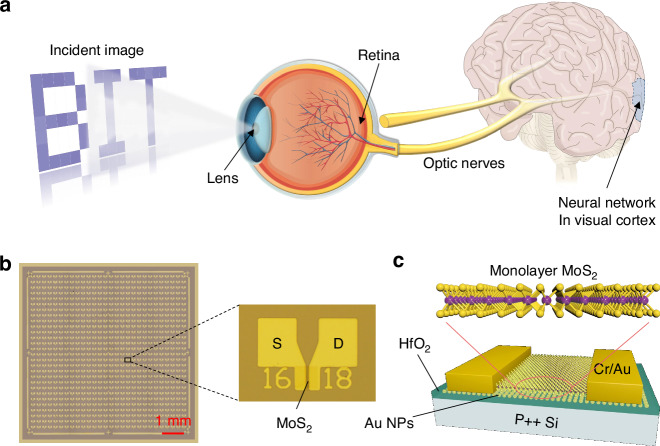
Human visual system and artificial synaptic devices array. **a** Schematic diagram of the human visual perception system, including neural networks in the human lens, hemispherical retina, optic nerve, and visual cortex. **b** Optical image of the 28 × 28 device array. **c** 3D schematic diagram of the artificial synaptic device structure based on the MoS_2_ floating-gate device

Visual perception is a pivotal sense for humans, with approximately 80% of the external information we receive coming through our eyes^29,49,50^. The process of visual perception begins in the retina, where neural cells detect and convert light signals into electrical spikes. These spikes are then transmitted through the optic nerve to the visual cortex located at the back of the human brain, as illustrated in Fig. 1a. The intricate process allows humans to perceive, interpret, and interact with their surrounding environment. Mirroring the biological process, the artificial optic-neural network composed of the 28 × 28 synaptic devices array (~0.7 × 0.7 cm^2^) can simply simulate the process of image recognition, as depicted in Fig. 1b. The detailed floating-gate structure of the device manufactured on the Si substrate is presented in Fig. 1c. Initially, a 20 nm HfO_2_ as the insulating layer was deposited on the substrate using atomic layer deposition (ALD), followed by the thermal deposition of 2 nm discontinuous Au film. The average size of the Au NPs was approximately 15 nm, as shown in Supplementary Fig. S1. The subsequent 10 nm HfO_2_ was deposited as the tunneling layer. Then, the monolayer MoS_2_ grown on the sapphire was transferred onto the pre-prepared silicon substrate using wet etching technology and transferred onto a prefabricated silicon substrate. As shown in the Supplementary Fig. S2, we used large-area wafer grade MoS_2_, which exhibited excellent uniform characteristics. The Raman spectra of the transferred MoS_2_ film in Supplementary Fig. S3 shows two characteristic peaks E^1^_2g_ at ~384 cm^−1^ and A_1g_ at ~404 cm^−1^, corresponding to the in-plane vibration and out-of-plane phonon coupling modes of MoS_2_, respectively. The ~20 cm^−1^ difference between two peaks indicates the monolayer properties of the MoS_2_ film prepared by the CVD method^36,51–53^. Additionally, the atomic force microscopy (AFM) image reveals a smooth MoS_2_ surface without significant defects or contamination, with the channel material thickness measured to be ~0.48 nm (Supplementary Fig. S4). After the standard UV lithography, reaction ion etching and electrodes (Cr/Au, 5/35 nm) deposition, the MoS_2_ devices array with source-drain terminals were patterned. The meticulously designed artificial synaptic network not only embodies the principles of human visual perception but also paves the way for advanced applications in artificial intelligence and neuromorphic computing.

**Fig. 2:**
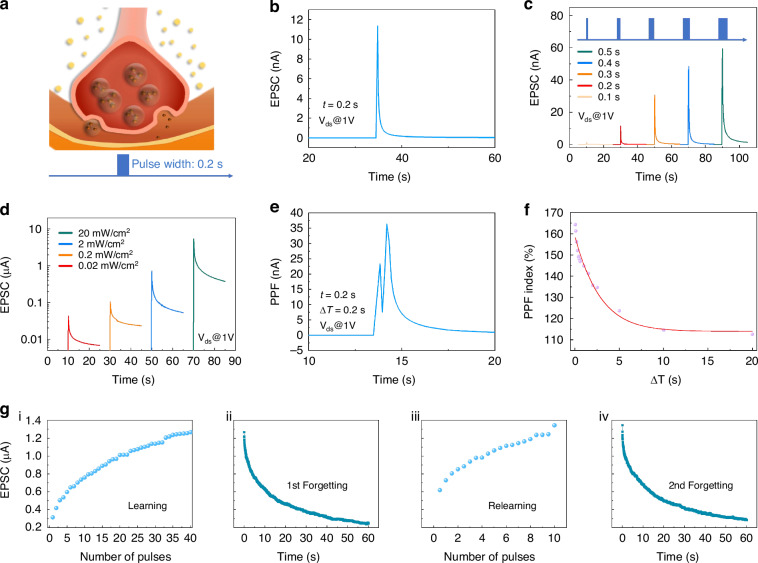
Plasticity of the artificial optoelectronic synapses. **a** Schematic diagram of biological synapses. **b** EPSC generated by pulsed laser-induced electrical artificial synapses, with a spike width of 0.2 s and a source drain voltage of 1 V. **c** EPSC generated by different widths of optical spikes. **d** EPSC generated by different optical power densities. **e** The synaptic transistor triggered by a pair of optical spikes with a duration of 0.4 s. **f** The PPF index of the EPSC plotted as a function of the spike interval (ΔT). **g** Simulation of the “learning-forgetting-relearning-forgetting” process using different optical spikes stimulation

Synapses represent the critical junctions within the human nervous system that connect two neurons for information transmission. Figure 2a depicts a schematic diagram illustrating the synaptic operation. Considering the hysteresis window shown in Supplementary Fig. S5, the Au NPs layer plays a pivotal role in electron capture in contrast to the devices without the floating-gate layer in Supplementary Fig. S6. There is no gate voltage applied in Fig. 2. Figure 2b portrays the generation of the postsynaptic current by the artificial synapse under the stimulation of the optical spike (λ = 520 nm, spike width t = 0.2 s, source/drain voltage V_ds_ = 1 V, optical power density P = 20 mW/cm^2^). The EPSC reached ~11 nA after a single learning session. The channel’s response to light serves as the analog to the input terminal of the presynaptic membrane, while the current in the channel between the source-drain electrodes can be defined as the EPSC of the postsynaptic membrane. Upon stimulation by the light spike, the MoS_2_ channel exhibited an increase in current due to photo-generated charges. A portion of these charges were subsequently captured by tunneling into the floating-gate layer. The removal of light facilitated the gradual release of captured charges, resulting in a progressive return of the current to its original state. Analogous to the biological neural signal transduction, the modulation of channel conductivity and the capture of charges by Au NPs facilitate synaptic weight and neurotransmitter transmission alteration, respectively. The variation in postsynaptic current with different spike widths (P = 20 mW/cm^2^, t ranging from 0.1 s to 0.5 s) was examined, revealing an increase in EPSC from 0.9 to 58 μA with incremental spike width, corresponding to the enhanced excitatory postsynaptic potential with prolonged stimulation, indicative of increased neurotransmitter release, as illustrated in Fig. 2c. Additionally, the postsynaptic current was measured across varying optical power densities (P ranging from 0.02 to 20 mW/cm^2^) in Fig. 2d, with the EPSC evidently increased from ~0.03 to ~5 μA with different optical density. Supplementary Fig. S7 shows the output curves of the device gated by different optical power densities ranging from 40 to 0 mW/cm^2^ with a step of −8 mW/cm^2^. The output curves show the characteristic of the device current decreasing as the optical power density weakens. Supplementary Fig. S8 displays the corresponding transfer curves of the floating-gate transistor under assorted optical power densities, corroborating the charges capture characteristics of the Au NPs floating-gate layer. The applied optical spike with different power can modulate the charge capture effect between the gate dielectric and the channel, thereby modifying the channel conductance of the device. Further, the frequency dependence of the EPSC was explored through the application of 20 optical spikes, revealing a gradual increase in EPSC with frequency escalation, as depicted in Supplementary Fig. S9. The experimental phenomena indicate the artificial synapse’s capability to simulate enhanced learning processes observed in biological organisms.

In the biological nervous system, PPF reflects the dynamic enhancement of postsynaptic current, closely related to learning, memory, and information-processing functions.

1 $${PPF\; index}={A}_{2}/{A}_{1}=\left[1+{C}_{1}{e}^{\left(-\frac{\triangle T}{{\tau }_{1}}\right)}+{C}_{2}{e}^{\left(-\frac{\triangle T}{{\tau }_{2}}\right)}\right]\times 100 \%$$

Here, A_1_ and A_2_ represents the peak intensities of EPSCs for the first and second spikes, respectively. C_1_ and C_2_ is separately defined as the original facilitation magnitudes of two phases, while *τ*_1_ and *τ*_2_ denote the corresponding relaxation times for C_1_ and C_2_. The PPF index is calculated to be ~160%, a value exceeding 100%, which signifies an augmented number of electrons induced by photons during the stimulation by the second spike, as illustrated in Fig. 2e, f. Moreover, the PPF index rapidly decrease from 160%, and then gradually approach the value of 100% as the inter-spike interval (ΔT) increases, which can be accurately modeled by a specific formula. The relaxation times, τ_1_ and *τ*_2_, were calculated ~87 and 4506 ms, respectively, values that are in alignment with the relaxation times observed in biological synapses.

The PPF effect, which amplifies the excited photocurrent and extends the decay period, enables the high-performance photoelectric synapses to simulate the typical “learning-memory-forgetting” behavior by two continuous optical spikes sequences with a 200 ms interval, as depicted in Fig. 2g. Initially, the device was irradiated with 40 spikes (λ = 520 nm, t = 0.2 s, ΔT = 0.2 s, V_ds_ = 1 V, P = 6 mW/cm^2^) to mimic the first learning process. The synaptic current started to increase to be saturated (EPSC ≈ 1.3 μA), mirroring the phenomenon of the human brain tending to become fatigued after repeated learning processes. Subsequently, the current decayed spontaneously to a moderate level after removing the light stimulus, which is consistent with the forgetting behavior over time. During the second learning process, only 20 identical spikes can reach the same current (synaptic weight) as in the first learning process, indicating that the relearning process requires less time. In addition, the decrease of synaptic weight during the second forgetting process (~0.29 μA) is weaker than the current during the first forgetting process (~0.22 μA), akin to the long-term memory ability of humans after repeated learning experiences. In the first learning process, some holes in the Au NPs layer are firmly occupied by electrons from the MoS_2_ channel through the Fowler-Nordheim tunneling under light excitation. The bounded electrons cannot quickly return to the channel in the first forgetting process, leading to that the current cannot decay to the original station. Subsequently, when the second light pulse was applied, due to the bounded electrons in the Au NPs layer coming from the first learning process, the better memory state (higher current) can be obtained (~0.29 μA > ~ 0.22 μA)^33,54,55^.

**Fig. 3:**
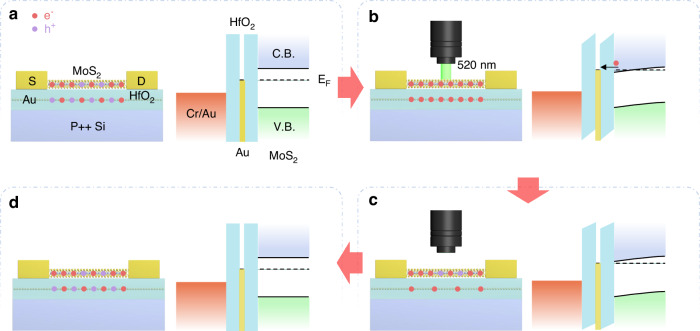
Schematic diagrams of the working mechanism of the optical synaptic transistor. The band energy of the device is regulated by the laser. Panels **a**–**d** separately demonstrates the working state of the device before applying laser, under 520 nm laser irradiation, after removing the laser, and slow recovery to the initial state

Figure 3 shows the detailed working principle of the optical synaptic device. Initially, the charges in the device were in a state of equilibrium, with the Fermi level of the MoS_2_ channel remained unaffected. Upon exposure to light, a significant number of electron-hole pairs were generated on the surface of the MoS_2_, leading to an accumulation of electrons within the channel and a consequent downward bending of the energy band^33,54,55^. Simultaneously, some induced electrons were captured by Au NPs floating-gate layer via the Fowler-Nordheim tunneling effect. After removing the optical spike, even though the dissipation of the enhancement effect, the electrons captured in the floating-gate layer were unable to immediately return back, resulting in a gradual depletion of electrons within the MoS_2_ channel. Subsequently, the electrons detained in the floating-gate layer slowly diffused back to the MoS_2_ channel layer over time, culminating in gradual recovery of PSC. At this stage, the PSC cannot quickly recover to its initial position due to electrons being bound by the floating-gate layer. Ultimately, the device reverted to its initial state following the complete depletion of the captured electrons. The electrical signals, sequentially pass through Au NPs floating-gate layer, HfO_2_ tunnel layer, and MoS_2_ channel, simulate presynaptic, synaptic, and postsynaptic process, respectively. And the entire process is similar to the behavior of EPSC in biological synapses.

**Fig. 4:**
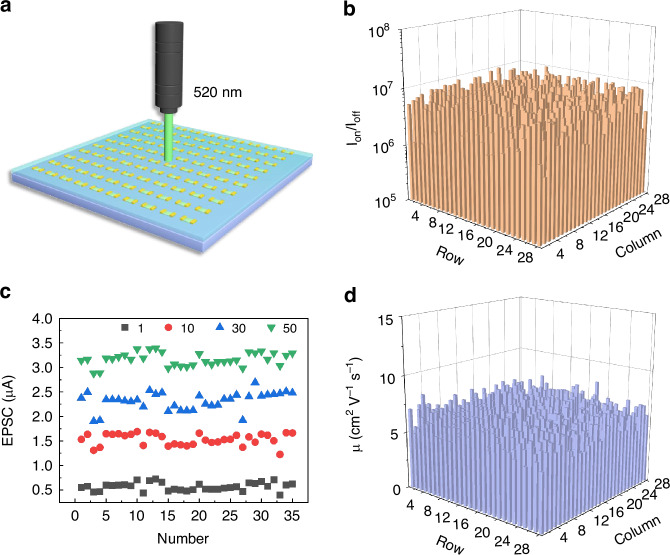
Excellent and uniform performance of 784 optoelectronic synapses. **a** Schematic diagram of the optical synaptic devices array illuminated by 520 nm light. **b** Statistical results of on/off ratios for 784 synaptic devices. **c** Statistical analysis of EPSC generated by the multi-pulse stimulation. **d** Statistical analysis of the mobilities of the devices array

Building on the exceptional synaptic performance exhibited by the single device, we fabricated a 28 × 28 artificial synaptic devices array on a single chip and undertook a comprehensive statistical analysis to assess their stability and uniformity. Figure 4a illustrates a schematic diagram of the artificial photoelectric synaptic devices array being illuminated by a 520 nm laser. Subsequent to the measurement of transfer curves across all devices, we derived statistical switch ratios and mobilities as shown in Fig. 4b, d. The consistent on/off ratios of the 28 × 28 devices array can exceed ~10^6^, with the average mobility of ~8 cm^2^V^−1^s^−1^, demonstrating the uniformity and indicating the potential for optic-neural network applications. Therefore, we selected a 5 × 7 synaptic devices array to simulate the process of biological synaptic learning and forgetting process. Figure 4c demonstrates the devices array to stimulate synaptic characteristics by applying varying numbers of consecutive laser spikes to induce multi-pulse EPSCs, further affirming the high homogeneity of the synaptic device array through statistical analysis. We calculated the means and the standard deviations of the on/off ratio, mobility, and EPSCs in Supplementary Table S1. Through numerical analysis of the means and standard deviations, it can be demonstrated that the device array has highly uniform performance.

**Fig. 5:**
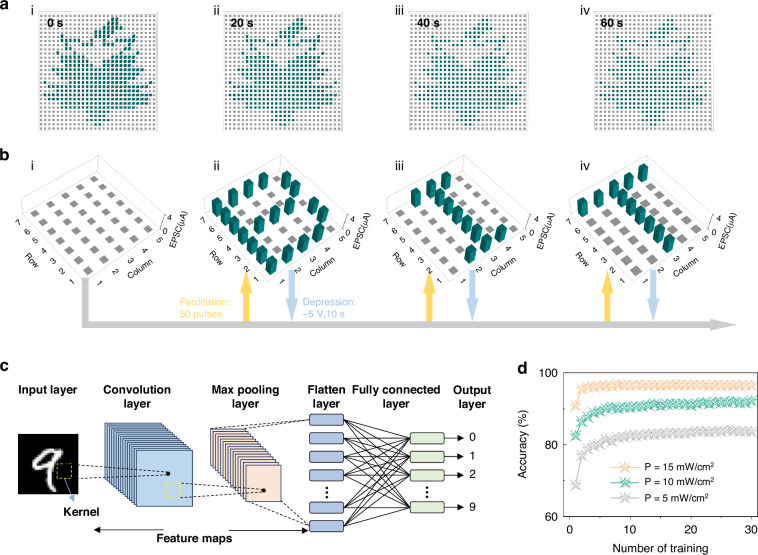
Synaptic devices array used for iconic memory and analog recognition. **a** Implemented 28 × 28 synaptic arrays as the trainable memory. The image of the emblem of Beijing Institute of Technology was input into the memory array using 50 spikes of laser irradiation with P = 20 mW/cm^2^, t = 0.2 s and ΔT = 0.2 s. **b** Applying different voltage spikes to write and erase the letter “B”, “I”, and “T”. **c** Schematic diagram of a simulated CNNs with an input layer that captures a 28 × 28-pixel image, a convolution layer with a 3 × 3 kernel, an average pooling layer followed by a fully connected layer and output layer. **d** The recognition accuracy of the visual signal stimuli for different training iterations under different light illumination

In our study, we successfully encoded the emblem of Beijing Institute of Technology into a 28 × 28 array of synaptic devices by applying 50 spikes with a power density of 20 mW/cm^2^, as illustrated in Fig. 5a. The spike width and interval time were uniformly set at 0.2 s and 0.2 s, respectively. By individually scanning the synaptic devices using optical signals, the image information of the emblem can be precisely stored in the synaptic devices array. The EPSCs recorded at time intervals of 0, 20, 40, and 60 s were approximately 3.3, 1.8, 0.6, and 0.1 μA, respectively. Furthermore, the results demonstrate the capability of the synaptic devices array to retain the image information for durations extending beyond 60 seconds is attributed to the slow discharge of charges from the floating-gate layer. The feature facilitates the perception and preservation of image information within the synaptic devices array. Moreover, to simulate the enhancement and inhibition of biological stimuli more intuitively, we applied promotion (50 optical spikes under the same conditions as those used for encoding the emblem, as shown in Fig. 5b (t = 0.2 s, ΔT = 0.2 s, V_ds_ = 1 V, P = 20 mW/cm^2^), and inhibition spikes (V_g_ = −5 V for 10 s) were applied, and the corresponding EPSCs of synaptic devices were recorded as ~3.2 μA. By varying the location of the applied light pulse, we were able to sequentially program the images of the letter “B”, “I”, and “T” into the device array, demonstrating the array’s ability to acquire and distinguish between different images. The capability underscores the potential of our synaptic devices array in the domain of optical information processing and storage, mirroring the dynamic and adaptable nature of biological synaptic functions.

To further explore the impact of light illumination on the learning capability of the device artificial synaptic array, the recognition of the National Institute of Standards and Technology (MNIST) handwritten digit database was simulated using a multilayer perceptron network, drawing on the measurements in Supplementary Fig. S10^56,57^. The schematic diagram in Fig. 5c can demonstrate CNNs based on model designed for recognizing a bunch of images of handwriting numbers from the MNIST dataset. The artificial visual neural network, which draws inspiration from the human retina, is structured in three layers: the photoreceptor layer, the intermediate neural cell layer (encompassing bipolar, horizontal, and amacrine cells), and the ganglion cell layer. The network architecture includes an input layer that captures a 28 × 28-pixel image, a convolution layer with a 3 × 3 kernel, a max pooling layer followed by a fully connected (FC) layer and an output layer (Fig. 5c). As shown in Fig. 5d, a recognition accuracy only of 83.1% was achieved under light illumination at a optical power density of 5 mW/cm^2^. Previous studies suggested that higher recognition accuracy correlates with larger ratios of maximum to minimum conductance (G_max_/G_min_) and good linearity^58–60^. The synaptic device under light illumination with the optical power density of 10 mW/cm^2^ and 15 mW/cm^2^ achieved larger G_max_/G_min_ and smaller nonlinearity (NL) (calculated from the long term depression/potentiation (LTD/LTP) curves (LTP: 40 optical spikes (t = 0.2 s, ΔT = 0.2 s), LTD: 40 electrical spikes (V_g_ = -3 V, t = 0.2 s, ΔT = 0.2 s)) and fitting of equations in Supplementary Fig. S10), culminating in enhanced recognition accuraciy of 91.3% and 96.5%, respectively. Therefore, this study demonstrates that adjusting the power density of light illumination can significantly enhance recognition accuracy, thereby offering a viable method for optimizing the performance of artificial visual neural networks.

## Conclusions

In summary, we design a highly uniform artificial visual neural network based on wafer-scale single-layer MoS_2_ floating-gated field effect transistors array, which is demonstrated as a 28 × 28 devices array within a 0.7 × 0.7 cm^2^ area. Each device exhibits stable and superior optoelectronic performance to simulate the plasticity of the visual synapse, thus allowing the integrated array of artificial synaptic devices to simulate human visual neural network. The biomimetic processes of perceiving, remembering, and forgetting visual signals are efficiently replicated through these artificial synaptic devices. The constructed artificial synaptic neural network offers high integration, stable uniformity, outstanding parallelism, and high efficiency. Through programming of optical signals and erasing images via electrical signals, it is anticipated that the ability to process optoelectronic signals in parallel will significantly augment the performance of future-generation computers. Furthermore, the synaptic weight updates regulated through the light signals has been leveraged for handwritten image recognition, achieving a high recognition accuracy of up to 96.5%. In order to avoid connection instability caused by complex circuits, we adopted the probe station to measure the properties of the devices. However, designing appropriate supporting circuits can help to make the testing more convenient and efficient, which is the direction we will further optimize. In short, the accomplishment underscores the network’s potential applicability in deep learning scenarios, highlighting its utility in advancing computational technologies.

## Methods

### Materials preparation

A custom-built, three-zone chemical vapor deposition (CVD) system was employed to synthesize monolayer MoS_2_ thin films. High-purity precursors, sulfur (S) (Alfa Aesar 99.9%) and molybdenum trioxide (MoO_3_) (Alfa Aesar 99.999%), were positioned in the first and second zones, correspondingly. A sapphire substrate was situated in the third zone. The temperatures for the three zones were precisely set to 125, 520, and 920 °C, respectively. Throughout the material growth phase, carrier gases argon (Ar) and an argon-oxygen mixture (Ar/O_2_) were utilized at flow rates of 110 sccm and 30 sccm, respectively. The pressure within the growth chamber was sustained at 1 torr, and a reaction time of approximately 50 min was necessary to achieve a fully covered film.

### Device fabrication

Firstly, a 20 nm HfO_2_ as the insulating layer was deposited on the substrate using the atomic layer deposition (ALD), followed by the thermal deposition of a 2 nm discontinuous Au film and a subsequent 10 nm HfO_2_ was deposited as the tunneling layer through the ALD method. The CVD-grown monolayer MoS_2_ was transferred onto a HfO_2_/Au/HfO_2_ substrate using PMMA (950 K, 5% in Anisole) as the support layer. Spin coat PMMA onto MoS_2_ film on sapphire substrate at 1500 rpm, and bake the substrate at 180 °C for 3 min to cure the PMMA. Put the sample in the 0.75 g/mL KOH solution and boil at 110 °C for 1.5 h, then wash it in the deionized water. Due to the etching effect of the KOH on the sapphire, the PMMA with MoS_2_ can separate from the substrate and float on the deionized water. The prepared Si substrate with HfO_2_/Au/HfO_2_ was used to pick the film up from the water. The substrate carrying the film should be left to air dry naturally for at least 8 h, and then baked at 80 °C to increase its bonding strength. Following the PMMA removal in acetone, photoresist (AZ-6130) was spin-coated onto the MoS_2_ film at 4000 rpm for 1 min, followed by baking the substrate at 110 °C for 3 min to remove the solvent. A 2.3 s UV exposure (∼30 mW/cm²) was defined the MoS_2_ channel pattern, and the reactive ion etching (RIE) was used to remove the excess MoS_2_. Subsequently, the photoresist (AZ-6130) was spin--coated onto the MoS_2_ film at a speed of 4000 rpm for 1 minute, and then the substrate was baked at 110 °C for 3 min to remove the solvent. A source drain electrode pattern with a 2.3 s UV exposure (approximately 30 mW/cm²) was defined, and thermal evaporation can realize to pattern Cr/Au (5 nm/35 nm) source-drain electrodes.

### Characterization

Raman spectroscopy was conducted using the Lab RAM HR-800 system with a 532 nm laser excitation. The thickness and surface uniformity of the MoS_2_ film were assessed with the Bruker Dimension XR Fast Scan Atomic Force Microscope (AFM). The electrical characteristics of the synaptic devices were performed using probe method combined with the Agilent B1500A Semiconductor Device Analyzer and Tektronix AFG310022 Arbitrary Waveform/Function Generator. Through connecting the common back gate and independent source-drain electrodes separately, we can measure the optical/electrical performance of each device one by one.

## Supplementary information

Supplementary Information
