# Supplementary material for: Large-scale high uniform optoelectronic synapses array for artificial visual neural network
Source: Microsyst Nanoeng. 2025 Jan 13;11:5. doi: 10.1038/s41378-024-00859-2 (PMC11731047; doi:10.1038/s41378-024-00859-2)
Supplement: Supplementary file 1 — Supplementary Information [file 41378_2024_859_MOESM1_ESM.docx]

**Supporting Information**

Large-Scale High Uniform Optoelectronic Synapses Array for Artificial Visual Neural Network

Fanqing Zhang^1,2,3^, Chunyang Li^1,2,3^, Zhicheng Chen^4,5^, Haiqiu Tan^5^, Zhongyi Li^1,2,3^, Chengzhai Lv^1,2,3^, Shuai Xiao^1,2,3^, Lining Wu^1,2,3^ and Jing Zhao^1,2,3,^*

1 State Key Laboratory of Explosion Science and Safety Protection, Beijing Institute of Technology, Ministry of Education, Beijing, 100081, China

2 School of Mechatronical Engineering, Beijing Institute of Technology, Beijing 100081, China;

3 Beijing Advanced Innovation Center for Intelligent Robots and Systems, Beijing Institute of Technology, Beijing 100081, China

4 Laser Micro/Nano Fabrication Laboratory, School of Mechanical Engineering, Beijing Institute of Technology, Beijing 100081, China

5 School of Mechanical Engineering, Beijing Institute of Technology, Beijing 100081, China

*Corresponding Author: jingzhao@bit.edu.cn


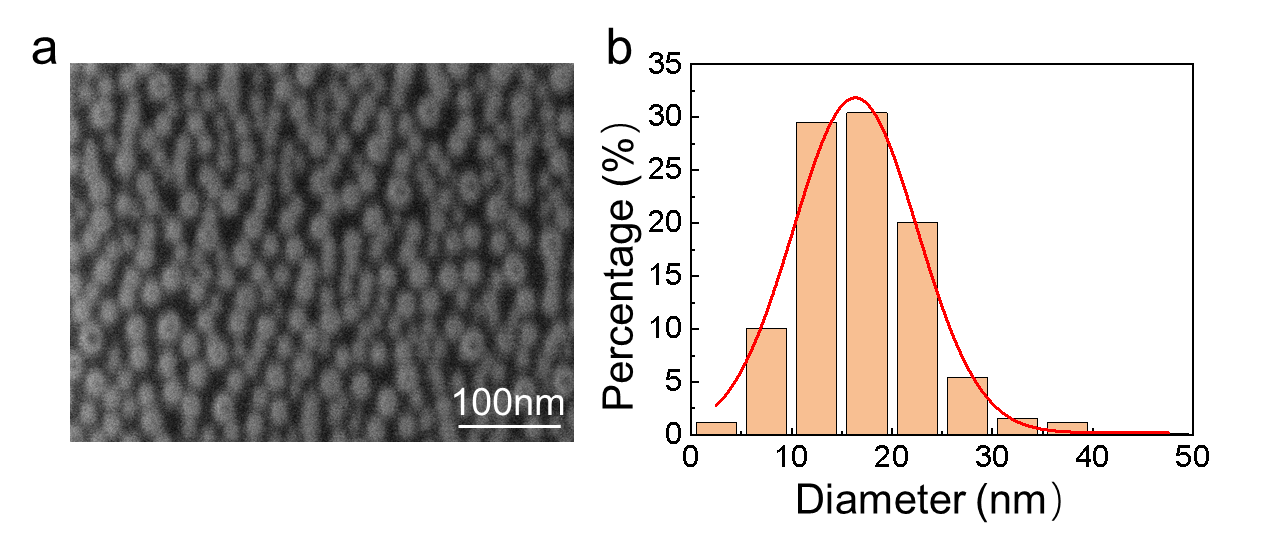


**Fig. S1. a** The scanning electron microscope (SEM) image of annealed Au NPs which average size is ~15 nm. **b** Percentage of diameter distribution of the Au NPs.


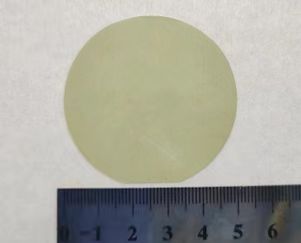


**Fig. S2. The optical image of the wafer-scale MoS_2_.**


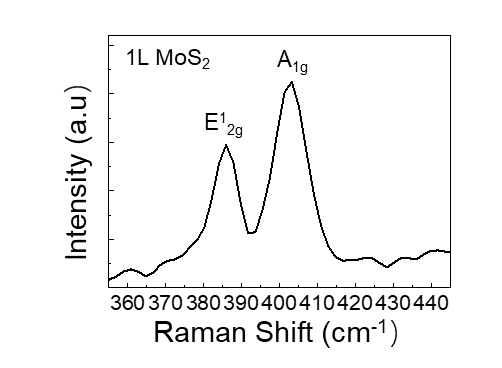


**Fig. S3. Raman spectrum of the MoS_2_ with the characteristic peaks at ≈ 384 cm^−1^ and ≈ 404 cm^−1^, confirming monolayer property of the utilized MoS_2_.**


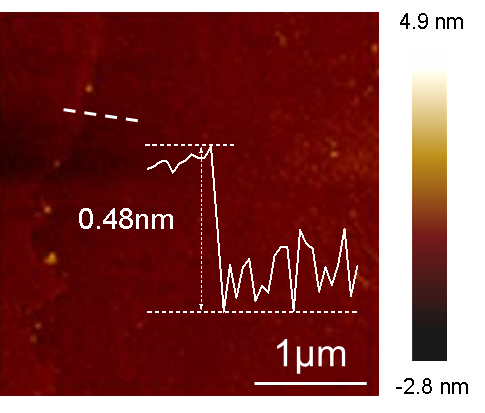


**Fig. S4.** **The atomic force microscope (AFM) image of the MoS_2_ channel.**


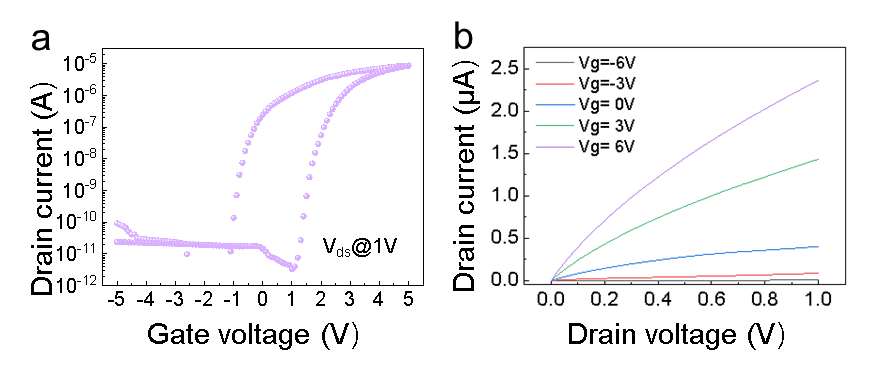


**Fig. S5. a** The transfer characteristics of the floating-gate synaptic transistor. **b** The output curves of the device with the back gate voltage ranging from -6 to 6 V with a step of 3 V.


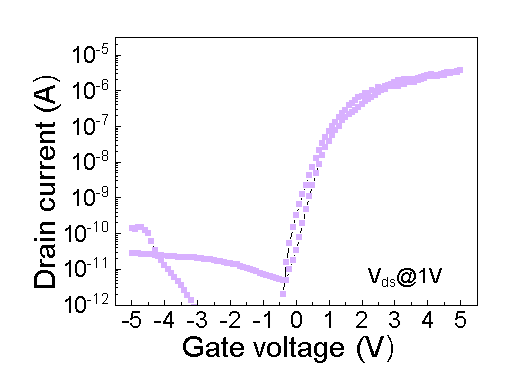


**Fig. S6. The transfer curve of the transistor without floating-gate layer.**


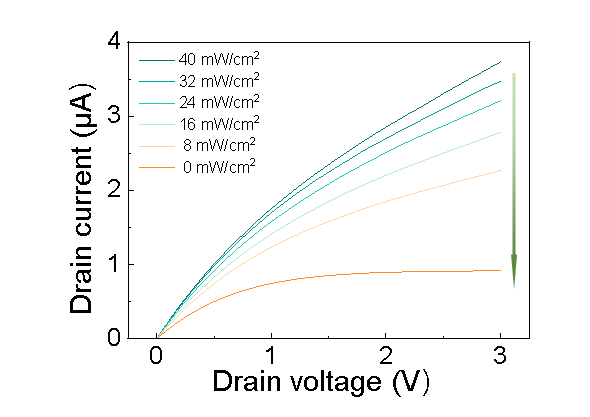


**Fig. S7.** **The output curves of the device gated by different optical power densities ranging from 40 to 0 mW/cm^2^ with a step of -8 mW/cm^2^.**


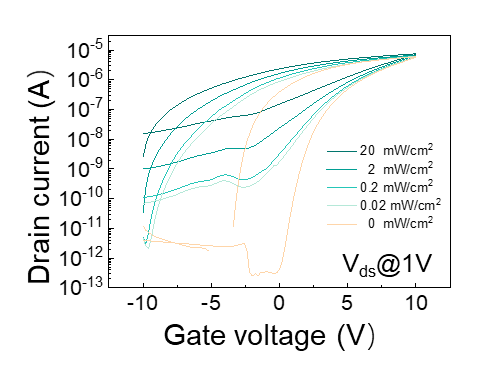


**Fig. S8. The transfer curves of the floating-gate synaptic transistor** **under different optical power densities excited by the 520 nm laser.**


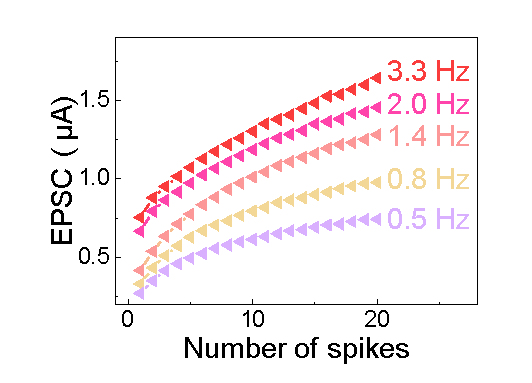


**Fig. S9.** **EPSC of the synaptic transistor with the laser spike of 0.2 s while the frequencies** **ranging from 0.5 to 3.3 Hz.**

**Table S1. The means and the standard deviations of the on/off ratio, mobility, and EPSCs.**

|  | Mean | Standard deviations | Total number |
| --- | --- | --- | --- |
| I_on_/I_off_ | 4.767E6 | 2.478E-6 | 784 |
| μ（cm^2^V^-1^s^-1^） | 6.250E1 | 7.276E-1 | 784 |
| EPSC_1_ （A） | 5.680E-7 | 8.312E-8 | 35 |
| EPSC_10_（A） | 1.534E-6 | 1.212E-7 | 35 |
| EPSC_30_（A） | 2.318E-6 | 1.826E-7 | 35 |
| EPSC_50_（A） | 3.165E-6 | 1.351E-7 | 35 |


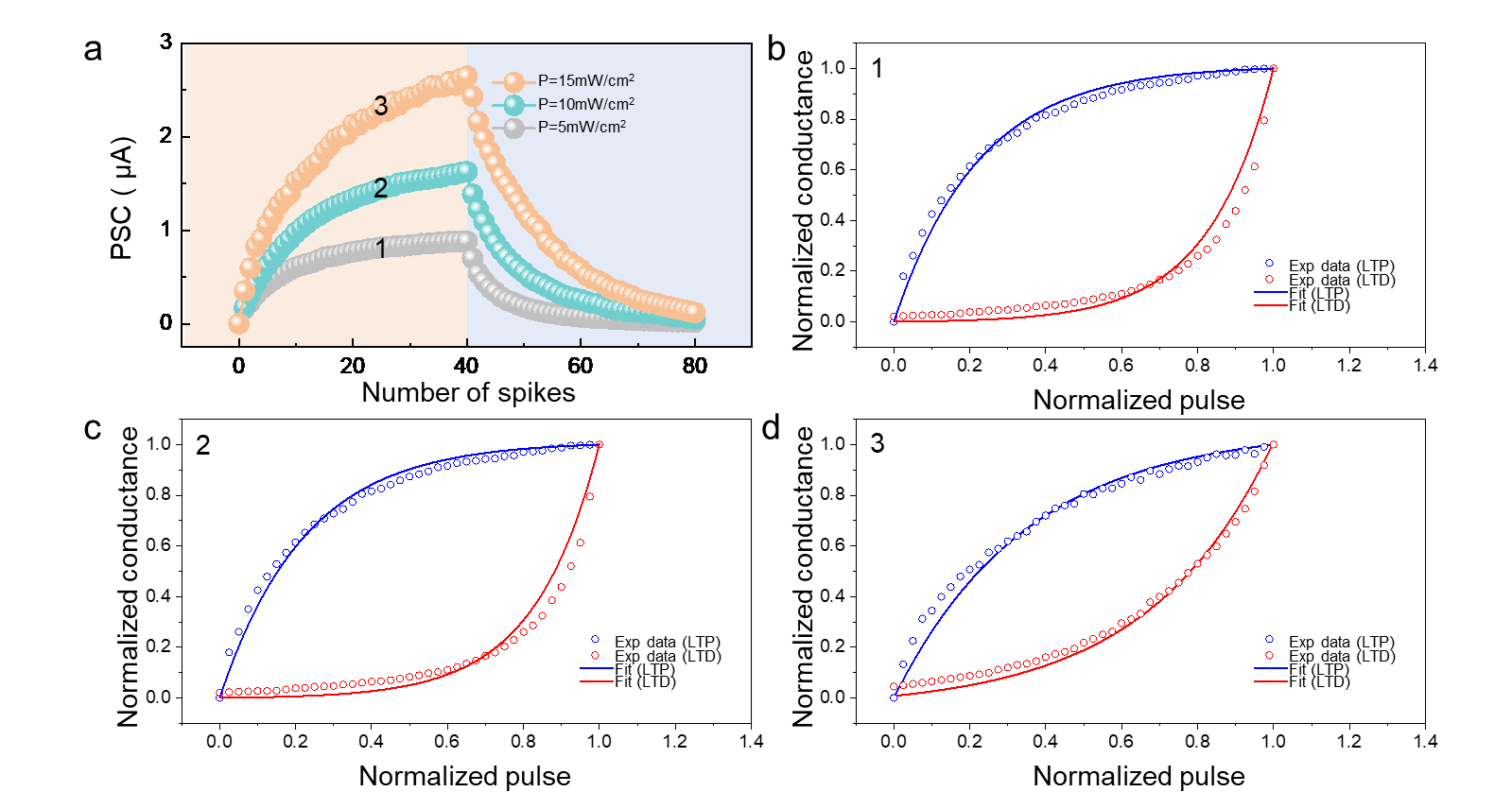


**Fig. S10. a** Plots of PSC as a function of spikes numbers through continuously applying series of optical spikes (t = 0.2 s, ΔT = 0.2 s) and electrical spikes (-3 V, t = 0.2 s, ΔT = 0.2 s) with different optical power density. **b, c, d** The weight update data of Floating-gate transistor is fitted based on the data from **a** with normalized amplitude in the plot of normalized conductance vs. the normalized number of pulses.

When multiple stimulus pulses are applied, the inhibitory behavior of synapses can be further enhanced. Fig. S10 shows the suppression of current by 40 electrical pulses under different lighting conditions.

The LTP/LTD behavior modes can refer to the following equation:

$$G=PSC/\left( U\times l \right) (1)$$

$$G_{LTP}=B\left[ 1-e^{\left( -\frac{p}{A} \right)} \right]+G_{min} (2)$$

$$G_{LTD}=-B\left[ 1-e^{\left( \frac{p-p_{max}}{A} \right)}+G_{max} \right] (3)$$

$$B=(G_{max}-G_{min})/\left[ 1-e^{\frac{-p_{max}}{A}} \right] (4)$$

In the above equations, G is the conductivity, PSC is the current intensity passing through the cross-section of the material, U (U= 1 V) is the potential difference perpendicular to the direction of the current, and l (l= 10 μm) is the length of the channel. G_LTP_ and G_LTD_ are defined as the observed conductivity values in the potentiation and depression curves, respectively. G_max_ and G_min_ correspond to the maximum and minimum conductivity values. The G_max_/G_min1,2,3_ values of ~ 242, 463 and 772 can be calculated at different optical power densities of 5,10,15 mW/cm^2^ in Fig. S10. p represents the number of the applied spikes, while p_max_ represents the maximum value that can be obtained. A and B are the fitting parameters, where A represents the magnitude of nonlinearity present in the potentiation and depression curves. The normalized A values and their corresponding nonlinearities (NL) in Table S1 can be calculated from 3 fitting curves at different optical power densities of 5,10,15 mW/cm^2^ in Fig. S10.

**Table S2. LTP and LTD behaviors of floating-gate MoS_2_ artificial synapses.**

|  | G_max_/G_min_ | Nonlinearity parameter (A_LTP_/A_LTD_) | Nonlinearity (NL_LTP_/NL_LTD_) | Recognition accuracy |
| --- | --- | --- | --- | --- |
| PSC_1_ | 242 | ~0.22/~-0.17 | 5.7371/-7.4257 | 83.1% |
| PSC_2_ | 463 | ~0.3/~-0.23 | 4.2057/-5.4874 | 91.3% |
| PSC_3_ | 772 | ~0.35/~-0.34 | 3.6038/-3.7101 | 96.5% |
